# Highly stable and immunogenic CMV T cell vaccine candidate developed using a synthetic MVA platform

**DOI:** 10.1038/s41541-024-00859-3

**Published:** 2024-03-30

**Authors:** Marcal Yll-Pico, Yoonsuh Park, Joy Martinez, Angelina Iniguez, Mindy Kha, Taehyun Kim, Leonard Medrano, Vu H. Nguyen, Teodora Kaltcheva, Shannon Dempsey, Flavia Chiuppesi, Felix Wussow, Don J. Diamond

**Affiliations:** Department of Hematology and Transplant Center, City of Hope National Medical Center, Duarte, CA USA

**Keywords:** Live attenuated vaccines, Viral infection, Herpes virus, Viral infection, Translational research

## Abstract

Human cytomegalovirus (CMV) is the most common infectious cause of complications post-transplantation, while a CMV vaccine for transplant recipients has yet to be licensed. Triplex, a multiantigen Modified Vaccinia Ankara (MVA)-vectored CMV vaccine candidate based on the immunodominant antigens phosphoprotein 65 (pp65) and immediate-early 1 and 2 (IE1/2), is in an advanced stage of clinical development. However, its limited genetic and expression stability restricts its potential for large-scale production. Using a recently developed fully synthetic MVA (sMVA) platform, we developed a new generation Triplex vaccine candidate, T10-F10, with different sequence modifications for enhanced vaccine stability. T10-F10 demonstrated genetic and expression stability during extensive virus passaging. In addition, we show that T10-F10 confers comparable immunogenicity to the original Triplex vaccine to elicit antigen-specific T cell responses in HLA-transgenic mice. These results demonstrate improvements in translational vaccine properties of an sMVA-based CMV vaccine candidate designed as a therapeutic treatment for transplant recipients.

## Introduction

Human cytomegalovirus (CMV) is a prototypic herpesvirus family member^1,2^. It is an opportunistic human pathogen with prevalence depending on age, socioeconomic status, and geographical location, reaching 100% in some locates^3^. While CMV infection is usually asymptomatic in healthy individuals, CMV can cause severe disease in the developing fetus and individuals with compromised immunity^4–6^. Moreover, CMV is the most prevalent and serious infectious complication post-transplantation in both hematopoietic stem cell transplant (HCT) and solid organ transplant (SOT) recipients^6,7^. Although CMV is well-known as a leading cause of complications in vulnerable target groups, currently there is no CMV vaccine licensed, neither for the prevention of congenital CMV infection nor for the prevention of CMV infection post-transplantation. Therefore, the development of a CMV vaccine for at-risk individuals is considered of critical importance to address an unmet medical need.

Modified Vaccinia Ankara (MVA) is an antigen delivery system widely used to develop infectious disease and cancer vaccines^8,9^. The vector is the basis of JYNNEOS, the only FDA-approved smallpox/monkeypox vaccine^10,11^. MVA is a highly attenuated poxvirus vector that acquired multiple genome alterations as a result of 570 virus passages in chicken embryo fibroblasts (CEF)^12,13^. A late block in virus assembly has made it incapable of productively infecting most mammalian cells, including human cells^14^. This translates into an excellent safety profile for vaccine development as MVA allows DNA replication and antigen expression without productive virus propagation in the vaccine recipient. MVA has a highly versatile expression system with a large capacity to incorporate heterologous DNA that allows simultaneous expression of multiple antigens to elicit potent humoral and cellular immunity^8,9,15,16^. The combination of these features makes MVA an ideal choice for a recombinant vaccine development strategy.

Different CMV antigens have been associated with protective immunity, stimulating notable levels of CD8+ and CD4 + T cell subsets, which aligns with the goal of developing a CMV vaccine for transplant recipients^1,17^. This includes the tegument protein pp65 (UL83) and the gene expression regulators immediate-early 1 (IE1, UL123) and immediate-early 2 (IE2, UL122)^18^. While IE1 has been shown to mainly stimulate CD8 + T cells, IE2 stimulates a vigorous CD8+ and a smaller CD4 + T cell memory response and pp65 stimulates significant levels of both CD8+ and CD4 + T cell subsets^17^. Therefore, these three antigens are excellent candidates to include in a vaccine designed to boost the immunity of the recipient against CMV infection or reactivation in the early stages post-transplant.

Several multiantigen MVA-based CMV vaccine candidates for the prevention of congenital infection and transplant recipients have been developed by our group^18–23^. This includes Triplex, a CMV vaccine candidate based on CMV antigens pp65 and a fusion construct composed of IE1 and IE2 (IEfusion) designed to boost CMV-specific T cell immunity post-transplantation^18^. Triplex has been shown to be safe and to elicit potent antigen-specific T cell responses in healthy and immunocompromised individuals. It was also shown to reduce CMV viremia and the need for antiviral therapy in a Phase 2 clinical trial in HCT early post-transplant recipients^24–27^. Triplex is the most advanced clinically evaluated CMV vaccine for transplant recipients and is being further tested in multiple Phase 2 clinical trials. However, while pp65 was stably expressed, IEfusion antigen expression was found to decrease upon extended virus passages in CEF. Therefore, the projected large-scale production and manufacturing of Triplex is limited due to this antigen expression instability. Additional stability research done by our group pointed to the IE2 fragment as the main source of instability of the IEfusion antigen. IE2 protein has been described as having DNA binding functions and to act as a direct roadblock to transcription elongation^28^. Furthermore, it has been demonstrated that substituting specific amino acid residues in a particular region of its C-terminus sequence inhibits DNA binding, abolishes autorepression, and impacts transactivation^29^. Our team conducted further research on this particular IE2 region using Triplex and different Triplex-like constructs, highlighting the importance this region has for stability within the specific MVA vector expression context.

We recently developed a system to rapidly generate recombinant MVA vectors based on a fully synthetic poxvirus platform, allowing reconstitution of synthetic MVA (sMVA) that is virtually identical to parental MVA in terms of genome constitution, replication properties, and immunogenicity^30^. Using sMVA, we developed multiantigen COVID-19 vaccine COH04S1, which demonstrated efficacy in pre-clinical animal models and has shown to be safe and immunogenic in Phase1/2 clinical trials^30–33^. In this study, we describe the development of T10-F10, a highly stable and immunogenic second-generation Triplex vaccine candidate using the sMVA platform. This vaccine candidate utilizes several modifications compared to the original Triplex to enhance vaccine stability, including altered antigen insertion, codon-optimized CMV antigen sequences, and an acquired mutation in the mentioned IE2 C-terminus specific region critical for antigen stability within the vector expression context (M361I). In this report, we present the stability assays and immunogenicity studies results obtained for T10-F10 and describe the modifications that were applied to the antigen sequences and to the vector construction to enhance vaccine stability over extended viral passages to enable an efficient and scalable large-scale production strategy.

## Results

### Construction of T10, an sMVA vector co-expressing CMV antigens IE1, IE2 and pp65

**Fig. 1:**
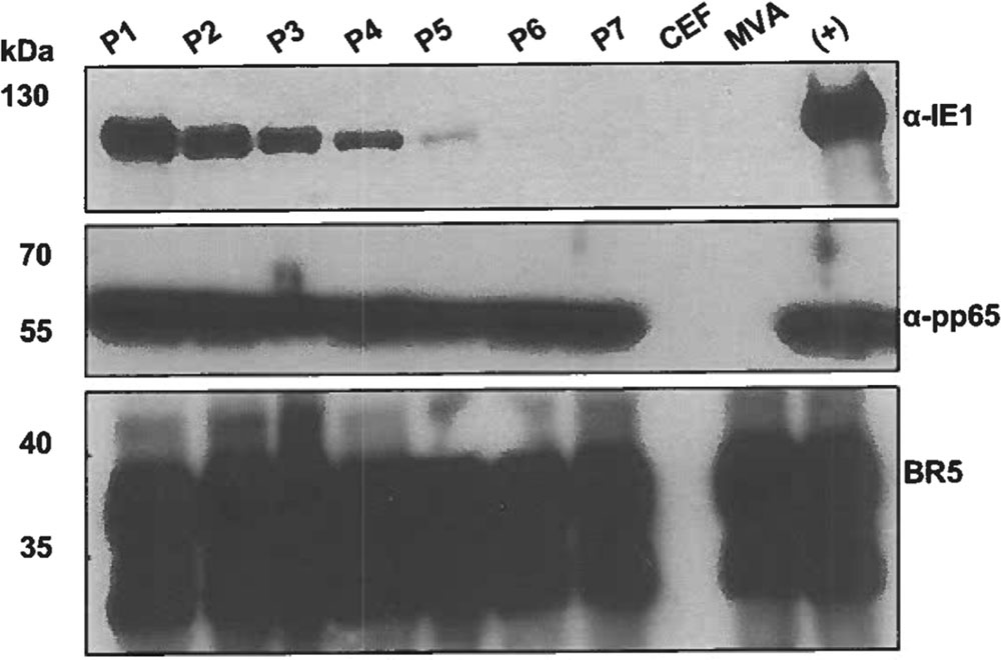
Triplex protein expression stability. WB analysis of the CMV proteins and the MVA control protein expression of Triplex passaged up to passage 7 in CEF. IEfusion was probed using an anti-IE1 mouse mAb (p63-27). Pp65 was probed using an anti-pp65 mouse mAb (28-103). Vaccinia virus BR5 was probed using an anti-pp65 rat mAb (19C2). “CEF” lane corresponds to uninfected cells. “MVA” lane corresponds to cells infected with the empty MVA vector. “(+)” corresponds to cells infected with virus stock used to generate clinical lots of Triplex. kDa: kilodalton.

While Triplex has shown to elicit robust T cell responses to all three CMV antigen in multiple clinical trials^24–27^, extended stability experiments in CEF showed limited expression stability of the IEfusion antigen sequence inserted into the MVA deletion 2 (Del2) site, whereas stable expression was observed for the pp65 antigen sequence inserted into the MVA Deletion 3 (Del3) site (Fig. 1).

To overcome the instability of the IEfusion antigen, we rederived Triplex using a vaccine design based on the sMVA platform. Different sequence modifications were applied to the CMV antigen sequences to achieve superior genetic and protein expression stability in the context of sMVA vector replication. Because the pp65 antigen sequence could be stably propagated in the original Triplex vaccine vector, it was unaltered in the new sMVA-based vaccine design. In contrast, several modifications were applied to the original IEfusion sequence. First, IEfusion was split into the original fragments IE1-exon4 (UL123), and IE2-exon5 (UL122) to insert them separately into different insertion sites. We hypothesized that the reduced length of heterologous DNA within the same insertion site will reduce the risk of introducing mutations. As in the original Triplex vaccine, both IE1 and IE2 antigens lacked the nuclear localization signal (NLS), causing directed localization in the cytoplasm. Another sequence modification applied to the IE1 and IE2 antigen sequences was a codon optimization strategy named “4nt”. It takes advantage of the redundancy of the genetic code to remove multiple repetitions of the same nucleotide without altering the amino acid sequence. This modification likely improves RNA polymerase activity preventing polymerase slippage and introduction of point mutations^34^. The modified antigen sequences were named IE1-4nt and IE2-4nt.

**Fig. 2:**
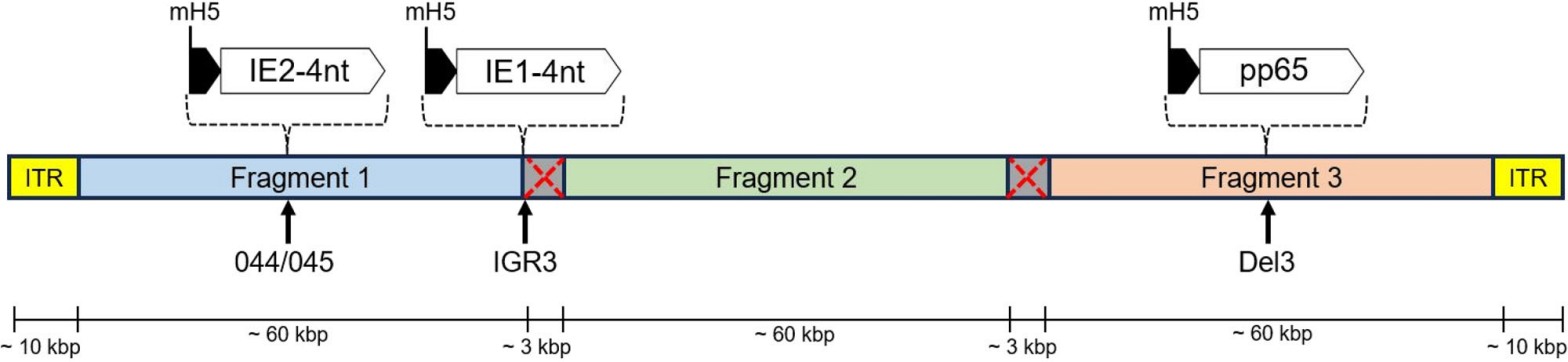
sMVA-T10 construct design. Schematic representation of the T10 vaccine construct reconstituted using three sMVA fragments. The insertion of the IE2-4nt, IE1-4nt, and pp65 CMV antigens sequences in each corresponding insertion site (44/45, IGR3, Del3) as well as the approximate length of the sMVA fragments, the overlapping homologous sequences for recombination between fragments 1 and 2 and fragments 2 and 3 (indicated as grey boxes with a red cross), and the inverted terminal repeats (ITR) is indicated. kbp kilobase pairs.

The CMV antigen sequences were inserted into the sMVA platform by bacterial artificial chromosome (BAC)-based recombination methods in *E. coli*^35,36^. The sMVA platform consists of three fully synthetic sub-genomic ~60 kbp DNA fragments (F1-F3) with ~3 kbp overlapping homologous regions in between F1-F2 and F2-F3, with F1 and F3 containing the ~10 kbp inverted terminal repeats (ITRs). These sMVA fragments allow virus reconstitution following co-transfection into permissive baby hamster kidney (BHK) cells and subsequent infection with Fowl pox virus (FPV) as a helper virus to initiate viral transcription^30^. While the IE1-4nt antigen sequence was inserted into the intergenic region (IGR) between MVA 064 L and 065 L (IGR64/65, also known as IGR3) within sMVA fragment F1, the IE2-4nt antigen sequence was inserted into the IGR between MVA 044 L and 045 L (IGR44/45, referred to as 44/45) within sMVA fragment F1 and the pp65 antigen sequence was inserted into the Del3 site within sMVA fragment F3. Different insertion sites and antigen compositions were also tested, but these resulted in either inefficient virus reconstitution or unstable constructs where IE2 showed limited expression stability over viral passages. sMVA F1 with IE2-4nt in 44/45 and IE1-4nt in IGR3, unmodified sMVA F2, and sMVA F3 with pp65 in Del3 was found to be the better combination. Each CMV antigen sequence was inserted together with a modified H5 promoter (mH5) to drive the expression during early and late MVA replication phases^23,25^. The modified sMVA fragments F1 and F3 with the respective inserted antigen sequences and the unmodified sMVA fragment F2 were co-transfected into BHK cells, followed by infection by FPV strain TROVAC as a helper virus^37^, resulting in virus reconstitution of vaccine construct T10 (Fig. 2). T10 was then expanded (small-scale expansion) and titrated.

### Derivation of T10 virus isolates evidencing instability of the IE2-4nt sequence

T10 was plaque-purified to obtain more homogeneous viral isolates. Ten different T10-derived isolates were obtained, named T10-A9/B4/F1/F2/F3/F4/F10/G4/H9/H10. The IE2-4nt gene was sequenced in all ten plaque-purified isolates with special interest in its C-terminus region as previous experiments pointed to IE2 and this specific region as the main source of instability in the various antigen-insertion site combinations tested. The original non-mutated IE2-4nt sequence was observed in only two out of ten isolates (T10-B4/F2), while a substitution of the original methionine for an isoleucine in the amino acid position 361 (M361I) was detected in three out of ten isolates (T10-A9/F1/F10). In all three cases this amino acid substitution was caused by the same mutation, a single nucleotide substitution (G to T) in the third nucleotide of the codon corresponding to the amino acid position 361. The other five isolates showed different aberrant IE2 sequences, including reading frame shifts and mutations in the promoter. These results indicated instability of the original IE2-4nt sequence and spontaneous selection of a specific amino acid substitution (M361I).

### The M361I mutation in T10-derived virus isolates stabilizes IE2 antigen expression

**Fig. 3:**
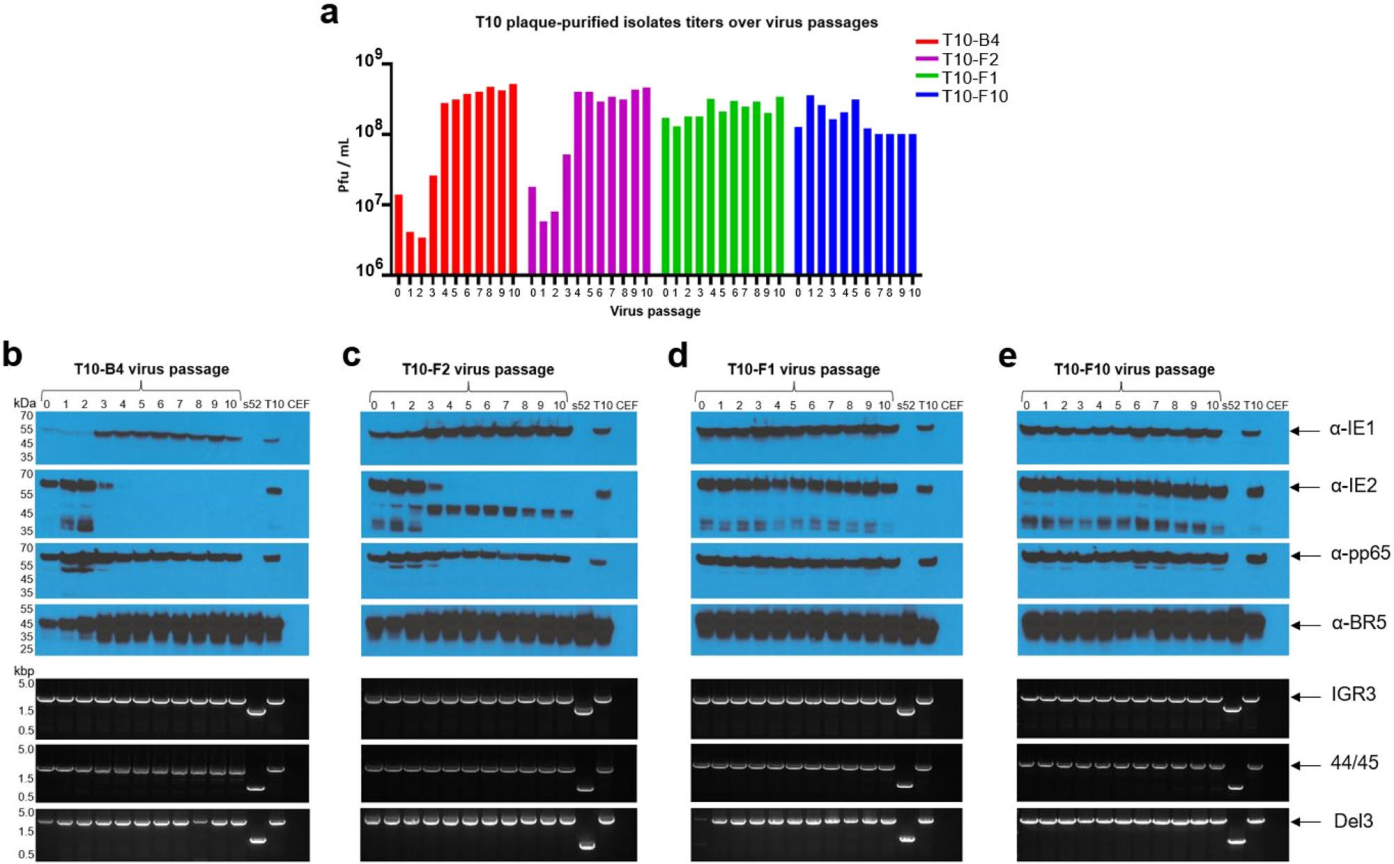
Antigen stability of non-mutated and IE2 M361I-mutated T10-derived virus isolates. T10-derived virus isolates containing non-mutated (B4 and F2) or M361I-mutated (F1 and F10) IE2 antigen sequences were blind passaged ten times in CEF and evaluated for antigen stability by PCR and WB. **a** virus titer. Given are the virus titer (pfu/mL) of the T10 plaque-purified isolates (T10-B4 in red, T10-F2 in purple, T10-F1 in green and T10-F10 in blue) during the ten virus passages in CEF. **b**–**e** WB and PCR analysis of T10-B4 (**b**), T10-F2 (**c**), T10-F1 (**d**), T10-F10 (**e**) passaged up to passage 10 in CEF. Upper panels show WB for the CMV proteins and BR5. IE1 ( ~ 55 kDa) was probed using an anti-IE1 mouse monoclonal antibody (mAb) (p63-27)^48^. IE2 ( ~ 63 kDa) was probed using an anti-IE2 mouse mAb (2.9.5)^49^. Pp65 (~65 kDa) was probed using an anti-pp65 mouse mAb (28-103)^50^. Vaccinia virus BR5 ( ~ 43 kDa) was probed using an anti-pp65 rat mAb (19C2)^51^. Lower panels show PCR analysis of the CMV gene sequences in the corresponding insertion sites. Expected PCR products are: 2,936 bp for IE1-4nt in IGR3, 2,219 bp for IE2-4nt/IE2-4nt-M361I in 44/45 and 2,882 bp for pp65 in Del3. “s52” and “T10” lanes correspond to cells infected with the empty sMVA vector and the parental T10 respectively. “CEF” lanes correspond to uninfected CEF cells. kbp: kilobase pairs; kDa: kilodalton.

To investigate the role of the M361I mutation in the IE2-4nt sequence stability, we compared the antigen stability of non-mutated and mutated T10-derived isolates following ten virus passages in CEF. For this comparison, we selected two virus isolates containing the original unmutated IE2-4nt sequence (T10-B4 and T10-F2) and two virus isolates containing the M361I mutation (T10-F1 and T10-F10). Notably, relatively low virus titers were observed for the original stocks (passage 0) of the non-mutated T10-B4 and T10-F2 isolates and a significant decrease in the titers of these isolates was observed at passages 1 and 2 (Fig. 3a). A recovery of the T10-B4 and T10-F2 virus titers was observed at passage 3, followed by a marked increase in T10-B4 and T10-F2 virus titers at passage 4, reaching virus titers that significantly exceeded the virus titers observed at passage 1. T10-B4 and T10-F2 virus titers remained stable throughout passages 5–10. In contrast, M361I-mutated T10-F1 and T10-F10 virus isolates showed elevated virus titers for the original stocks and the titers remained stable throughout the entire ten virus passages.

PCR analysis of the antigen insertion sites was performed to investigate the genetic integrity of CMV antigens over ten virus passages. This revealed the same PCR products throughout the ten virus passages for all four virus isolates, showing the expected PCR products in all cases and the absence of any additional PCR products that would suggest instability (Fig. 3b–e). Western Blot (WB) analysis was performed to investigate CMV antigen expression following ten virus passages. BR5, an MVA constitutively expressed protein^23^, was included in the WB analysis as a vector control. While the non-mutated T10-B4 and T10-F2 virus isolates showed stable expression of the pp65 and IE1 proteins over 10 virus passages, these isolates showed a dramatic decrease in expression of IE2 at passage 3 and a complete loss of IE2 antigen expression at passage 4 (Fig. 3b, c). Notably, loss of the IE2 antigen expression appeared to coincide with an enhanced IE1 and BR5 expression and with the marked increase in virus titers at passage 4 of the T10-B4 and T10-F2 virus isolates, indicating that loss of the non-mutated IE2 antigen expression results in dramatically increased replication fitness. In contrast, the M361I-mutated T10-F1 and T10-F10 virus isolates showed stable antigen expression of all three CMV antigens throughout the ten virus passages (Fig. 3d, e). The detected antigen protein products for these mutated isolates were expressed equally throughout the ten virus passages. Given that the M361I mutation in the IE2-4nt sequence is the only known difference between T10-B4/F2 and T10-F1/F10 virus isolates, these results highlight the importance of M361I mutation for the vaccine stability.

The IE2 gene was sequenced in samples from passage 10 of the four T10-derived isolates analyzed to assess how the IE2-4nt and IE2-4nt-M361I sequences changed during virus passage. The sequencing results revealed no changes in the M361I-mutated IE2-4nt gene sequence of the T10-F1 and T10-F10 isolates from passage 0 to passage 10, confirming the stability of the IE2-4nt-M361I sequence in these isolates. In contrast, the IE2-4nt sequence of both T10-B4 and T10-F2 isolates showed frameshift mutations resulting in truncated IE2 proteins, consistent with the IE2 instability observed by WB. Frameshift mutations in the T10-B4 and T10-F2 isolates were also detected for virus passage 4, which coincided with the loss of the IE2 antigen expression observed for these isolates by WB. These sequencing results may help explain the poor IE2 protein expression that the non-mutated isolates showed in the WB results on Fig. 3b, c.

**Fig. 4:**
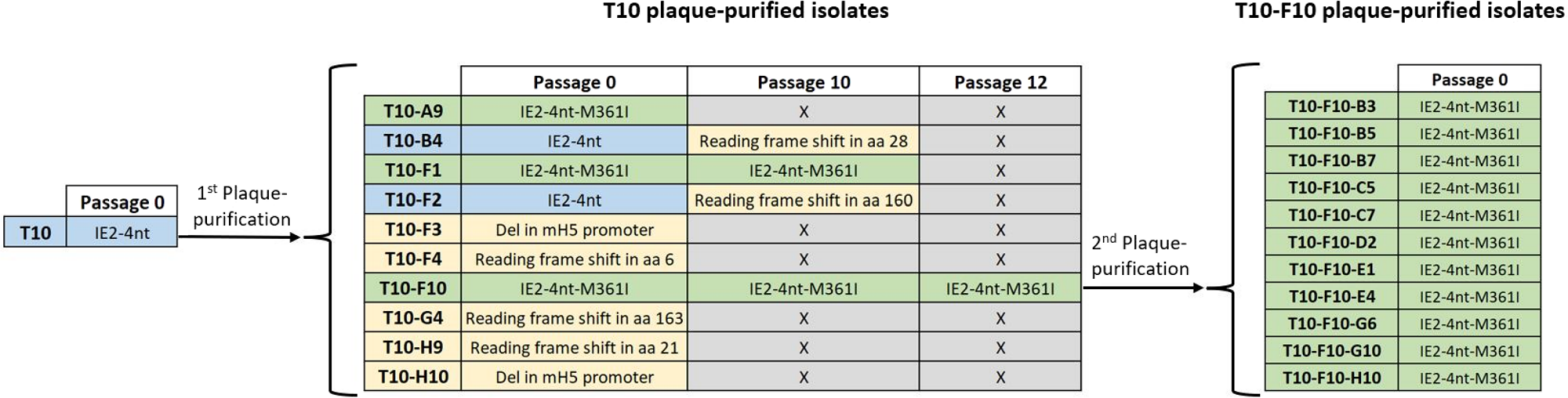
IE2 gene sequencing of the T10 and T10-F10 plaque-purified isolates. Schematic representation of the plaque-purification processes of T10 and T10-F10 where the IE2 gene sequencing results are indicated for the original stock (passage 0) of all isolates, passage 10 of the isolates used for the stability comparison assay and passage 12 of T10-F10. Sequencing results in blue boxes indicate original IE2-4nt sequences, green boxes indicate mutated IE2-4nt-M361I sequences and yellow boxes indicate aberrant sequences. Grey boxes with an “X” indicate sequencing not performed.

To further assess the importance of the M361I mutation for the IE2 antigen stability, the M361I-mutated T10-F10 isolate underwent an additional round of plaque-purification and the IE2 gene sequence of the eleven isolates obtained (T10-F10-B3/B5/B7/C5/C7/D2/E1/E4/G6/G10/H10) was evaluated by sequencing analysis. All eleven isolates showed identical IE2 gene sequence to the parental T10-F10, with all encoding for the M361I mutated sequence. The IE2 gene sequencing results, schematized in Fig. 4, corroborate the importance that the M361I mutation has for the IE2 antigen stability within the MVA vector expression context. Once this mutation is spontaneously acquired it is stably maintained, resulting in a stable viral population.

### T10-F10 provides stable antigen expression in CEF and AGE1.CR.PIX cells

**Fig. 5:**
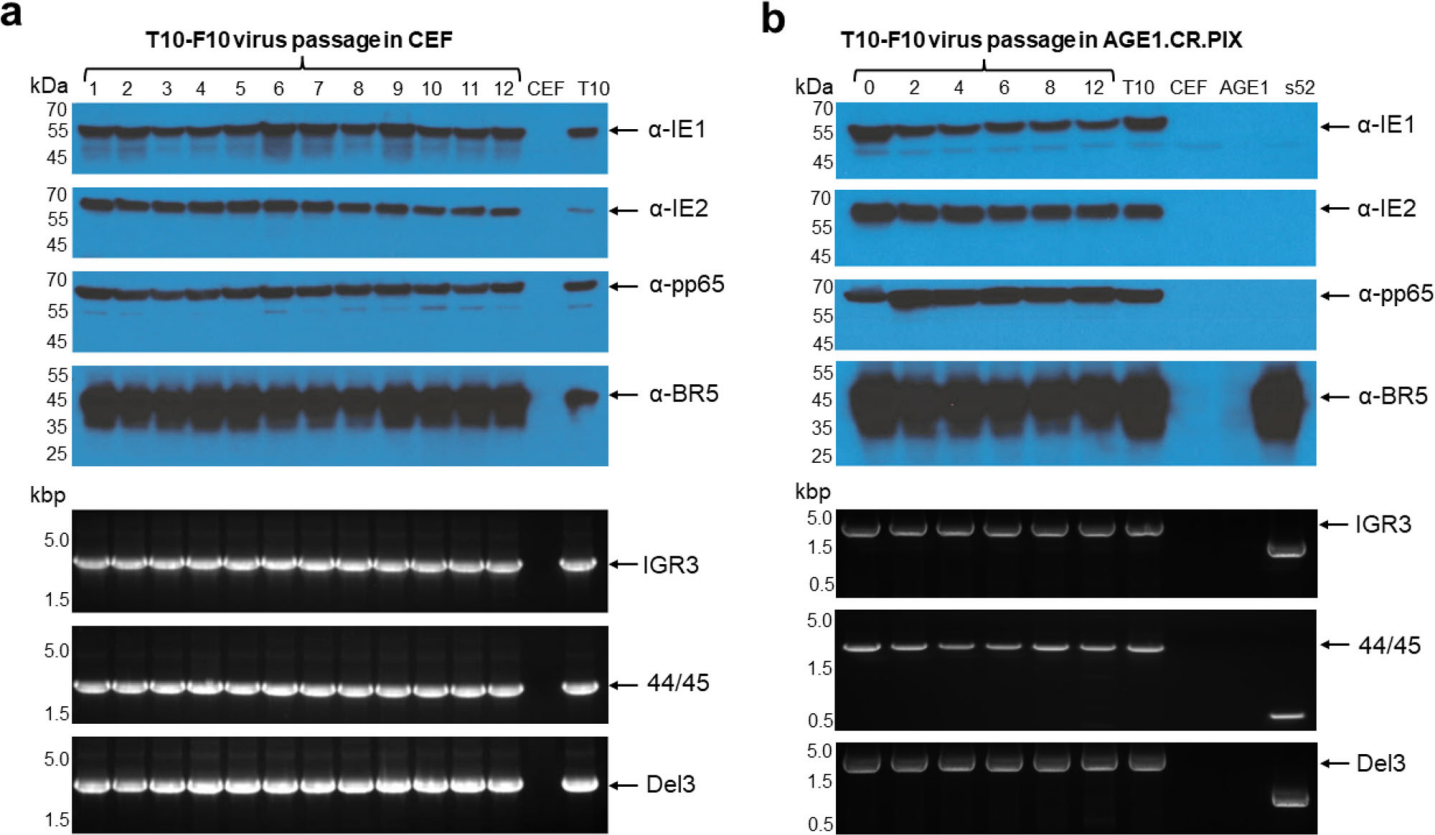
T10-F10 antigen stability in CEF and AGE1.CR.PIX cells. T10-F10 was blind passaged twelve times in CEF (**a**) and passaged twelve times in AGE1.CR.PIX (**b**) cells and evaluated for antigen stability by PCR and WB. Upper panels correspond to WB analysis of the CMV proteins and the MVA vector control expression. IE1 ( ~ 55 kDa) was probed using an anti-IE1 mAb (p63-27)^48^. IE2 ( ~ 63 kDa) was probed using an anti-IE2 mouse mAb (2.9.5)^49^. Pp65 (~65 kDa) was probed using an anti-pp65 mouse mAb (28-103)^50^. Vaccinia virus BR5 ( ~ 43 kDa)^51^ was probed using an anti-pp65 rat mAb (19C2). Lower panels correspond to PCR analysis of the CMV genes sequences in the corresponding insertion sites. Expected PCR products are: 2,936 bp for IE1-4nt in IGR3, 2,219 bp for IE2-4nt-M361I in 44/45 and 2,882 bp for pp65 in Del3. “s52” and “T10” lanes correspond to cells infected with the empty sMVA vector and the parental T10 respectively. “CEF” and “AGE1” lanes correspond to uninfected CEF and AGE1.CR.PIX cells. kbp: kilobase pairs; kDa: kilodalton.

Of the two stable IE2-4nt-M361I virus isolates, T10-F10 was selected for further genetic insertion and protein expression stability investigation. First, we reassessed the stability of T10-F10 following twelve virus passages in CEF, which confirmed the genetic and protein expression stability of all three CMV antigens in the T10-F10 virus isolate (Fig. 5a). Additionally, we investigated the CMV antigen stability of T10-F10 over twelve virus passages in suspension culture in the AGE1.CR.PIX duck cell line, which is a GMP compliant suspension cell line enabling large-scale manufacturing in bioreactors^38,39^. PCR analysis of the antigen sequence insertion sites confirmed the expected PCR products without detection of non-specific bands or loss of band intensity consistent with genetic stability. In addition, WB analysis revealed consistent protein expression with no apparent degradation products throughout the twelve virus passages for all three CMV antigens (Fig. 5b). These results demonstrate genetic and protein expression stability of T10-F10 following extensive virus propagation in CEF and AGE1.CR.PIX cells, two cell types known to allow manufacturing of MVA clinical products. To further confirm the stability of the M361I mutation, the IE2 gene from passage 12 of T10-F10 was fully sequenced, corroborating that the IE2-4nt-M361I sequence remained stable from passage 10 to passage 12 (Fig. 4). No other mutations were detected in the sequence.

### T10-F10 and original Triplex elicit comparable CMV-specific immunity in HLA-transgenic mice

T10-F10 was large-scale expanded and ultra-purified, and its immunogenicity was compared with the original Triplex vaccine in HLA-transgenic mouse models. This included HHDII mice, which are transgenic mice with the mouse H-2Db α3, transmembrane, and cytoplasmic domains but expressing human HLA MHC-I allele A*0201 (HLA-A2)^40^ and HLA-B7 mice, which are transgenic mice expressing the human HLA MHC-I allele B*0702 (HLA-B7)^41^. HLA-A2 and B7 transgenic mice were immunized twice in four weeks interval with 1 × 10^7 pfu/ml of T10-F10, Triplex, or s52, an sMVA vector without antigens inserted used as control to verify that the detected immune response was specifically induced by the CMV antigens and not as result of unspecific stimulation by the vector itself. At one week after the booster immunization, CMV-specific T cells were evaluated by ELISpot using IE1, IE2, and pp65 peptide libraries as well as pp65 and IE1-specific peptides of immunodominant CD8+ T cell epitopes. Specifically, HLA-A2-restricted pp65- and IE1-specific immunodominant epitope peptides pp65 495–503 (NLVPMVATV) and IE1 316-324 (VLEETSVML) and HLA-B7-restricted pp65-specific immunodominant epitope peptide pp65 265-275 (RPHERNGFTVL) were used.

**Fig. 6:**
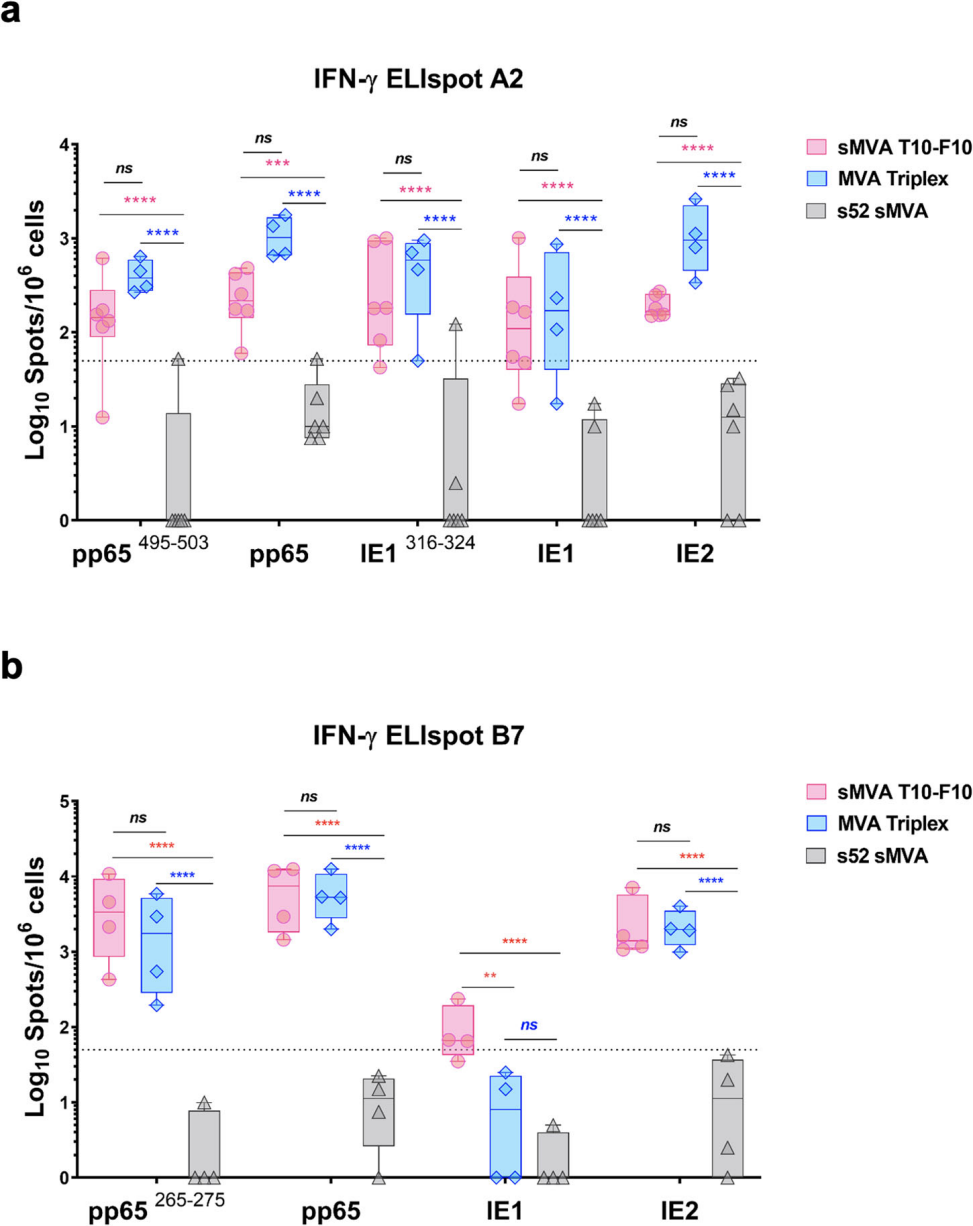
Potent stimulation of CMV-specific IFNγ-expressing T cells by T10-F10 in HLA-transgenic mice. **a** HLA-A2 transgenic mice ELISpot analysis of IFNγ-secreting cells following stimulation with CMV pp65, IE1 and IE2 peptide libraries and pp65 and IE1 peptides containing the HLA-A*0201-restricted pp65- and IE1-specific immunodominant epitopes (pp65 495–503 and IE1 316–324). **b** HLA-B7 transgenic mice ELISpot analysis of IFNγ-secreting cells following stimulation with CMV pp65, IE1 and IE2 peptide libraries and pp65 peptide containing the HLA-B*0702-restricted pp65-specific immunodominant epitope (pp65 265–275). Two-way ANOVA with Tukey’s multiple comparison test was used to calculate significance of the difference between the groups. Data is presented as mean values ± SD; **0.01 <*p* < 0.001, ***0.001 <*p* < 0.0001, ****p < 0.0001; ns not significant.

The ELISpot results for immunized HLA-A2 mice (Fig. 6a) showed robust CMV-specific T cell responses elicited by T10-F10 and Triplex. T cell responses elicited by Triplex tended to be higher than those elicited by T10-F10. Mainly for pp65 495-503 peptide and pp65 and IE2 libraries where a 2, 4 and 6-fold difference was observed respectively. However, these differences were not statistically significant. The mean IFNγ levels obtained for T10-F10 and Triplex were, respectively, 200 and 416 for pp65 495–503 peptide (*p* value = 0.319), 261 and 1118 for pp65 library (*p* value = 0.1371), 404 and 544 for IE1 316-324 peptide (*p* value = 0.8207), 246 and 306 for IE1 library (p value = 0.9376) and 193 and 1220 for IE2 library (*p* value = 0.1205). Therefore, no statistical differences were observed between the T cell responses elicited by T10-F10 and Triplex in HLA-A2 mice. On the other hand, IFNγ levels elicited with either the peptide libraries or the immunodominant peptide epitopes in T10-F10 or Triplex-vaccinated HLA-A2 mice were all significantly elevated compared to those measured in s52-vaccinated control mice. T cell responses elicited in HLA-B7 mice (Fig. 6b) by T10-F10 and Triplex were comparable when IFNγ levels were measured following stimulation with the pp65 or IE2 libraries while a 2-fold higher response was measured in T10-F10 for the pp65 265-275 peptide stimulation. The mean IFNγ levels obtained for T10-F10 and Triplex were, respectively, 4488 and 2390 for pp65 265-275 peptide (p value = 0.6035), 7189 and 6288 for pp65 library (p value = 0.9994) and 2741 and 2239 for IE2 library (p value = 0.9998). No statistical difference was detected in the IFNγ levels produced to these stimuli between T10-F10 and Triplex groups whereas significant statistical difference was detected when comparing them to the s52 group. However, the IE1-specific responses measured following stimulation with IE1 library in HLA-B7 mice vaccinated with T10-F10 were 10-fold higher and statistically superior to those measured in Triplex-vaccinated HLA-B7 mice. The mean IFNγ levels obtained were 101 for T10-F10 and 11 for Triplex (*p* value = 0.004) for IE1 library and, in this case, no statistical difference was detected between Triplex and s52 groups. These results indicate that the T10-F10 vaccine candidate elicits potent CMV-specific T cell responses in HLA-A2 and HLA-B7 transgenic mice that are comparable to those elicited by the original Triplex vaccine.

## Discussion

In this study, we demonstrate the development of second-generation Triplex vaccine candidate, termed T10-F10, providing stable expression of IE1, IE2, and pp65 antigens and potent stimulation of CMV-specific T cell responses to boost T cell immunity against CMV in transplant recipients. This vaccine candidate has been generated on the fully synthetic sMVA vaccine platform previously developed by our lab which has already demonstrated to efficiently and rapidly produce recombinant MVA vectors from chemically synthesized DNA to develop synthetic poxvirus-based vaccines expressing SARS-CoV-2 antigens to prevent the infection^30–32^. Because the original Triplex vaccine was found to provide limited antigen stability compromising its utility for large-scale production strategy for commercialization, we reformulated the vaccine design applying different strategies focused on the genetic insertion and protein expression stability improvement. These strategies included splitting the IEfusion protein from the original Triplex design^18^ into the two original fragments IE1 and IE2, codon-optimizing IE1 and IE2 sequences by applying the 4nt modification, using different vector insertion sites and maintaining the mH5 promoter to boost the antigen expression without sacrificing stability. Moreover, we found that the spontaneous acquisition of a methionine to isoleucine amino acid substitution in the amino acid position 361 (M361I) of the IE2 sequence was critical for the stable propagation and expression of the IE2 antigen. Using the sMVA platform and applying all these modifications we developed the vaccine candidate T10-F10, that elicits a robust T cell response against CMV in HLA-A2 and HLA-B7 transgenic mice while stably maintaining the inserted CMV antigens over more than ten virus passages.

While the precise reasons for the IEfusion instability in the original Triplex vaccine remain unclear, prior unpublished studies with different antigen-insertion site combinations pointed to the IE2 antigen fragment as a cause for the loss of the IEfusion antigen expression in Triplex. This profound instability of the parental IE2 antigen sequence was confirmed by sequencing analysis of the T10-derived virus isolates. While just 20% of T10-derived isolates expressed the parental IE2-4nt gene sequence, 50% showed totally aberrant IE2 sequences that wouldn’t translate into the appropriate protein and 30% contained the M361I mutation, that in all cases was caused by the same exact nucleotide transversion event. The M361I-mutated isolates maintained the rest of the sequence intact, with no other mutations or alterations. In addition, virus isolates containing the non-mutated IE2-4nt antigen sequence, including T10-B4 and T10-F2, suffered from abrupt and complete IE2 antigen loss during early phase following virus passage in CEF, which coincided with a dramatic increase in virus titers indicative of increased replication fitness. Further sequence analysis confirmed frameshift mutations within the IE2-4nt gene sequence of the passaged T10-B4 and T10-F2 virus isolates that are consistent with the observed antigen loss. In contrast, virus isolates T10-F10 and T10-F1 containing the M361I mutation did not show genetic changes of the IE2 antigen sequence or insertion sites or a decrease of the IE2 antigen expression. These findings were consistent with the stability assay results of the parallel comparison of the isolates T10-B4/F2 and T10-F1/F10 (Fig. 3b–e). T10-F1/F10 isolates, containing the M361I mutation, demonstrated genetic insertion and protein expression stability of all three CMV antigens over ten virus passages in CEF while unmutated IE2-4nt sequence of T10-B4/F2 isolates resulted in profound instability of IE2 expression. These results suggest that the non-mutated parental IE2 antigen has a strong repressive effect on the replication of the vector that results in antigen loss, while acquisition of the M361 mutation results in elimination of this repressive effect and specific stabilization of IE2.

Why the M361 mutation stabilizes the IE2 antigen sequence and protein expression within the sMVA-vectored T10-F10 virus isolate remains unclear, although several observations may provide an explanation. CMV IE2 protein has been described to have DNA binding functions, being able to act as a direct roadblock to transcription elongation^28^. In addition, there is a specific region within the C-terminus of IE2 which assumes a zinc finger conformation where amino acids substitution inhibits DNA binding, abolishes autorepression, and impacts transactivation. This region is located between amino acid positions 428 and 452^29^. However, as indicated before, we removed the NLS sequence from our IE2 design, so it lacks the first eighty-three amino acids of the N-terminus, meaning that original amino acid positions 428 and 452 are now positions 345 and 369 in the IE2 sequence. Therefore, the position where we detected the stabilizing M361I mutation falls within this region associated with DNA binding functions. Different Triplex-like constructs were analyzed in depth by our group, some of the analysis with special emphasis on investigating the 345-369 region of IE2 which indicated the key role that certain amino acid substitutions within this region could have on improving stability. These analyses are consistent with the pattern we observed in the present study since all isolates not containing the M361I mutation evolved to having mutations in the mH5 promoter or frameshift mutations that were located in amino acid positions before the 345-369 region.

The transcription elongation roadblock function of IE2 has been described to occur due to the binding of IE2 protein to a 14 nucleotide DNA consensus sequence: “CGTTTTGGAAAACG“^28^. This sequence can be found with just 1 mismatch (“CGATTTGGAAAACG”) in the sMVA sequence, specifically in the sMVA ORF MVA090R, that corresponds to an RNA polymerase subunit (rpo147). Given that situation, a transcription roadblock in MVA090R caused by IE2 could lead to decreased RNA polymerase production and to impaired replication fitness. These findings may suggest that the M361I mutation could eliminate this DNA binding function. Another mechanism in which the M361I mutation could be involved is the prevention of aberrant translation initiation by eliminating the original methionine at position 361 which might be acting as a cryptic start codon. The Kozak consensus sequence, being defined as “GCCGCCRCCATGG” where R correspond to A or G, plays a key role in the initiation of the translation process^42,43^. However, the Kozak sequence for this IE2 potentially cryptic start codon was found to be weak since its sequence “CCCTTCCTCATGG” did not match the defined consensus. We can’t yet provide a specific mechanism for why the M361I mutation improves stability in the specific vector expression context. Further studies would be needed and are beyond the scope of this work.

The extended stability assay performed with T10-F10 confirmed that this isolate is optimal for an efficient manufacturing strategy. It is genetically stable and shows strong CMV protein expression over twelve virus passages in both CEF and AGE1.CR.PIX cell lines. The importance of these results is that the candidate T10-F10 is stable in two different GMP compliant cell lines that can be used to produce MVA clinical products. This will allow an efficient large-scale production strategy, making T10-F10 a suitable candidate for possible commercialization.

T10-F10 elicited a strong T cell immune response in humanized transgenic mouse models that do not express murine Class I alleles^40,41,44^. The HHDII mice results demonstrate that CMV IE1, IE2 and pp65 antigens expressed by the sMVA vector are processed and immunologically recognized as we detected IFNγ production following stimulation with either IE1, IE2 or pp65 libraries or pp65 or IE1 peptides corresponding to HLA-A*0201-restricted pp65- and IE1-specific immunodominant epitopes. The T cell responses observed in HHDII mice for T10-F10 were in all cases comparable to those observed for Triplex, with no statistical significance in the differences, indicating a comparable immune response elicited by both vaccines. T10-F10 immunogenicity was tested in HLA-B7 mice following the same approach as with the HHDII mice in order to investigate the property of the candidate to be recognized in two different HLA contexts. Effective processing and immunological recognition of CMV antigens was also demonstrated in HLA-B7 mice immunized with T10-F10. IFNγ production levels detected following stimulation with either IE2 or pp65 libraries or a pp65 peptide corresponding to an HLA-B*0702-restricted pp65-specific immunodominant epitope were in all cases similar to the levels observed for the mice immunized with Triplex, with no statistical differences. Notably, in the case of the stimulation with the IE1 library the immune response elicited by T10-F10 was shown to be statistically superior to the response elicited by Triplex in HLA-B7 mice, although this may have been a result of the low responses in two of the Triplex immunized mice. Having assessed the immune response produced by the T10-F10-expressed CMV antigens solely with the evaluation of IFNγ production is a study limitation, although a strong correlation has been demonstrated in the past between IFNγ production and cytotoxic function in murine models^22,45,46^. These results demonstrate that our newly developed T10-F10 candidate is as immunogenic as original Triplex in transgenic mice models indicating as well that it can be efficiently processed by HLA-A2 and HLA-B7 alleles as is the case of original Triplex^18^. These facts support its utility as a possible future clinical candidate.

In summary, using our proprietary sMVA vaccine platform we have developed T10-F10, a highly stable and immunogenic sMVA-vectored CMV T cell vaccine candidate targeting transplant recipients. T10-F10 provides stable genetic insertion and expression of all three CMV antigens following extensive virus passaging in CEF and in suspension culture in AGE1.CR.PIX cells, indicating that T10-F10 can be stably propagated in cell substrates commonly used for manufacturing of MVA clinical products. Additionally, we show that the T10-F10 provides similar or even superior immunogenicity compared to the original Triplex vaccine to elicit antigen-specific T cell responses in HLA-transgenic mice. These results demonstrate that T10-F10 represents a stable and immunogenic vaccine that can be large-scale manufactured to boost CMV-specific T cell responses in patients that are in need, such as immunocompromised transplant patients.

## Methods

### Cell lines and helper virus

BHK-21 (CCL-10) cells were purchased from the American Type Culture Collection (ATCC) and cultured in minimum essential medium with Earle’s salts and L-glutamine (MEM) supplemented with 10% FBS, 1% sodium pyruvate, 1% non-essential amino acids and 1% penicillin-streptomycin. CEF cells were purchased from Charles River Laboratories (10100795) and cultured in MEM supplemented with 10% FBS and 1% penicillin-streptomycin. AGE1.CR.PIX cells were purchased from ProBioGen and cultured in CD-U7 medium supplemented with 1X GlutaMAX-1, 10 ng/mL of LR3 IGF-1 and 2 g/L of sodium bicarbonate. Virus stocks of the FPV used as helper virus for reconstitution were produced following propagation on CEF cells using FPV strain TROVAC from ATCC (VR-2553)^37^. FPV titers were evaluated on CEF cells by virus plaque determination.

### Construction of sMVA-T10 and CMV antigen insertion

The construction of the sMVA fragments was done as previously described^30^.CMV IE1, IE2 and pp65 antigen sequences were inserted into the corresponding sMVA fragments by *En passant* mutagenesis in GS1783 *E. coli* cells^35,36^. Transfer constructs were created using the pGEM-T-mH5 vector that we previously generated^20^. The transfer constructs were composed of the IE1, IE2 or pp65 antigen gene sequence with an upstream mH5 promoter region and a downstream Vaccinia transcription termination signal (TTTTTAT). IE1, IE2 and pp65 antigen sequences were based on the CMV strain AD169. The Triplex sequence^18^ was used as a template for the pp65 sequence and a codon optimized IEfusion-4nt sequence synthetized by GenScript was used as a template to generate the IE1-4nt and IE2-4nt sequences. The transfer constructs also contained a kanamycin resistance cassette with an adjacent I-SceI homing endonuclease restriction site and a flanking 50-bp gene duplication^21^. To insert the transfer constructs into the corresponding sMVA fragments via Red-recombination^35,36^, the transfer constructs were PCR-amplified by Phusion polymerase (Thermo Fisher Scientific) using primers that provided ~50 bp extensions for homologous recombination. Primers 5’-AAT TGT ACT TTG TAA TAT AAT GAT ATA TAT TTT CAC TTT ATC TCA TTT GAT TTT TAT AAA AAT TGA AAA TAA ATA CAA AGG TTC-3’ and 5’-ATT CCG AAA TCT GTA CAT CAT GCA GTG GTT AAA CAA AAA CAT TTT TAT TCC TAG TAT AAA AAG GCG CGC C-3’ were used to insert the IE1-4nt antigen sequence into the IGR3 insertion site. Primers 5’-GAA TAT GAC TAA ACC GAT GAC CAT TTA AAA ACC CCT CTC TAG CTT TCA CTA AAA ATT GAA AAT AAA TAC AAA GGT TC-3’ and 5’-ATA ATG TTT TTA TAT TAT ACA TGT TCT AAA AGA ATA ATC GAT ACA GTT TAC TAG TAT AAA AAG GCG CGC C-3’ were used to insert the IE2-4nt antigen sequence into the 44/45 insertion site. Primers 5’-TTG GGG AAA TAT GAA CCT GAC ATG ATT AAG ATT GCT CTT TCG GTG GCT GGT AAA AAA TTG AAA ATA AAT ACA AAG GTT C-3’ and 5’-ACA AAA TTA TGT ATT TTG TTC TAT CAA CTA CCT ATA AAA CTT TCC AAA TAC TAG TAT AAA AAG GCG CGC C-3’ were used to insert the pp65 antigen sequence into the Del3 insertion site. Underlined regions indicate sequences used to produce the ~50 bp extensions for homologous recombination.

The NucleoSpin Gel and PCR clean-up kit (Macherey-Nagel) was used to purify the amplified PCR products after they had been run at 100 V on a 1% agarose gel stained with ethidium bromide. Then, 100 ng of PCR product were electroporated at 15 kV/cm, 25 μF, and 200 Ω into 50 μL of recombination-competent GS1783 bacteria containing the corresponding sMVA fragment. The bacteria were resuspended in 1 mL of antibiotic-free Luria-Bertani (LB) medium and incubated for 2 hours at 32 °C and 220 rpm. After that, the bacteria culture was streaked onto LB agar plates with 30 μg/mL chloramphenicol and 30 μg/mL kanamycin and incubated at 32 °C for 2 days. Bacterial clones containing the sMVA fragments with the corresponding inserted CMV antigen sequences at the respective insertion sites were identified and selected by PCR and Restriction Fragment Length Polymorphism (RFLP). A I-SceI-mediated second Red-recombination reaction was used to remove the kanamycin resistance marker from the CMV antigen sequences. For that purpose, 100 μL of overnight culture of the selected bacterial clones were added to 900 μL of LB medium containing 30 μg/mL chloramphenicol and incubated for 2 h at 32 °C and 220 rpm. Afterwards, 1 mL of LB containing 30 μg/mL chloramphenicol and 2% L-arabinose was added to induce the expression of the I-SceI homing endonuclease enzyme and to induce a double-strand break at the 50 bp gene duplication. The bacteria cultures were incubated for 1 h at 32 °C and then, to induce the expression of the Red-recombination proteins and to mediate the removal of the kanamycin resistance marker by recombination of the 50 bp gene duplication regions, were transferred to a water bath where it was incubated for 30 min at 42 °C and 220 rpm. Finally, after an additional incubation of 2 h at 32 °C and 220 rpm, the bacteria cultures were streaked onto LB agar plates with 30 μg/mL chloramphenicol and 1% L-arabinose and incubated at 32 °C for 2 days. Bacterial clones carrying the sMVA fragments without the kanamycin marker from the inserted CMV antigen sequences were identified by PCR, RFLP and Sanger sequencing.

### sMVA-T10 reconstitution

The three sMVA plasmids (fragments 1-3 with the respective CMV antigen sequences inserted) were isolated from the bacteria by alkaline lysis^47^ and cotransfected into a well from a 6-well tissue plate cultured with 70% confluent BHK cells using Fugene HD transfection reagent (Roche) according to the manufacturer’s instructions. At 4 h post cotransfection, the BHK cells were infected with ~0.1–1 multiplicity of infection (MOI) of FPV (TROVAC) to initiate sMVA-T10 virus reconstitution. The BHK cells were grown for 2 days and then splitted in 1:2 ratio and grown for additional 2 days in a larger tissue culture format. This process was repeated over a period of 12 days when most of the cells showed signs of sMVA virus infection (characteristic MVA viral plaque formation and cytopathic effects (CPEs) indicating sMVA virus reconstitution). The infected BHK cells were then harvested, centrifugated at 1200 rpm for 5 min at room temperature and resuspended in MEM supplemented with 2% FBS, 1% sodium pyruvate, 1% non-essential amino acids and 1% penicillin-streptomycin. sMVA-T10 virus was then prepared by conventional freeze/thaw method (3 rounds) and 2 rounds of sonication (1 sec ON / 1 sec OFF for 2 min at 500 Watt, 20 kHz), resulting in the sMVA-T10 original virus stock.

### Expansion, ultra-purification and titration of viral stocks

To generate small-scale expanded viral stocks, BHK cells were seeded in five 150 mm cell culture dishes, grown to ~80–90% confluency, and infected at 0.1 MOI with the corresponding viral stock. Two days post infection, the plates were harvested, combined, centrifugated at 1200 rpm for 5 min at room temperature and resuspended in MEM supplemented with 2% FBS, 1% sodium pyruvate, 1% non-essential amino acids and 1% penicillin-streptomycin. The small-scale expanded stock was then prepared by freeze/thaw method (3 rounds) and 2 rounds of sonication (1 sec ON/1 s OFF for 2 min at 500 Watt, 20 kHz). To generate large-scale expanded and ultra-purified viral stocks, CEF cells were seeded in thirty-five 150 mm cell culture dishes, grown to ~80–90% confluency, and infected at 0.1 MOI with the corresponding viral stock. Two days post infection, the plates were harvested and combined, and the ultra-purified viral stock was prepared by 36% sucrose cushion ultracentrifugation and virus resuspension in 1 mM Tris-HCl (pH 9). Viral stocks were in all cases stored at −80 °C. Vaccinia polyclonal antibody (pAb) (9503-2057, Bio-Rad. Dilution 1:2000) was used for the titer determination of the viral stocks by immunostaining of viral plaques at 16–24 h post infection of ~80–90% confluent CEF cells infected with serial dilutions of the respective viral stock.

### Plaque-purification of viral stocks

To generate plaque-purified isolates from a viral stock, CEF cells were seeded in 96-well plates, grown to ~80–90% confluency, and infected at different pfu/plate (ranging from 5 to 50 pfu/plate) with the corresponding viral stock. Four to five days post-infection, wells containing a single viral plaque were harvested and prepared by freeze/thaw method (3 rounds) and 2 rounds of sonication (1 sec ON/1 sec OFF for 2 min at 500 Watt, 20 kHz) to obtain the plaque-purified isolates. Then, to expand the plaque-purified isolates, the isolates were propagated first for two days in 24-well plates and subsequently for 2 days in T-75 flasks and harvested and prepared as mentioned before after each propagation process by freeze/thaw and sonication methods.

### Virus passaging

To study the stability of the sMVA constructs in CEF cells over consecutive virus passaging, CEF cells were seeded in one 150 mm cell culture plate, grown to ~80–90% confluency, and infected at 0.1 MOI with the corresponding viral stock (“passage 0”). Two days post infection, the plate was harvested, centrifugated at 1200 rpm for 5 min at room temperature, resuspended in 1 mL of MEM supplemented with 2% FBS and 1% penicillin-streptomycin and prepared by freeze/thaw method (3 rounds) and 2 rounds of sonication (1 sec ON/1 sec OFF for 2 min at 500 Watt, 20 kHz), obtaining the “passage 1” viral stock. This process was repeated for each consecutive passage as blind passaging, infecting with 10 uL of the previous passage until passage 10 for T10-B4/F1/F2 and passage 12 for T10-F10. Finally, all passages were titrated as described. The MOI ranges used (0.005–0.6 MOI for T10-B4 passages, 0.007 – 0.6 MOI for T10-F2, 0.1–0.4 MOI for T10-F1 and 0.1–0.4 MOI for T10-F10) were determined based on the back-titration. The low MOI range values from T10-B4 and T10-F2 correspond to the first passages of each construct in which the non-mutated IE2 was expressed being the titers significantly low. To study the stability of the sMVA constructs in AGE1.CR.PIX cells over consecutive virus passaging, 1 × 10^8^ AGE1.CR.PIX cells in 25 mL of medium contained in a 250 ml Erlenmeyer flask were infected at 0.03 MOI with the corresponding viral stock (“passage 0”) and then incubated at 37 °C and 8% CO_2_ while shaking at 180 rpm. Two days post infection, cells were collected, centrifugated at 4000 g for 10 min at room temperature, resuspended in 1 mL of medium and prepared by freeze/thaw method (3 rounds) and 1 round of sonication (1 sec ON/1 s OFF for 2 min at 500 Watt, 20 kHz), obtaining the “passage 1” viral stock. Finally, the viral stock was titrated as described. This process was repeated for each consecutive passage until passage 12 for T10-F10. Virus prepared from all passages in CEF and AGE1.CR.PIX cells were characterized by PCR and WB.

### PCR analysis

To characterize the insertion of the CMV gene sequences in the respective MVA insertion sites of the sMVA viral stocks, CEF cells were seeded in 6-well plates, grown to ~80–90% confluency, and infected at 1 MOI with the corresponding sMVA viral stock. At 16–24 h post infection, cells were harvested and DNA was extracted using the Quick-DNA Miniprep Plus Kit (ZYMO RESEARCH) according to the manufacturer’s instructions. PCR reactions were performed with DreamTaq polymerase (Thermo Fisher Scientific) using primer pairs that target flanking regions of the respective insertion site. Primers 5′-AAC AAG TCC CAG ATT ACG AGC C-3′ and 5′-ATT TGA TAG CCT GGA AGC ACA AG-3′ were used to characterize the insertion of IE1-4nt in IGR3. Primers 5′-TCC ATT GTA GAT TGT TGA CCG-3′ and 5′-ATA CAT ACC ATC GAC ATC CAT TAG C-3′ were used to characterize the insertion of IE2-4nt/IE2-4nt-M361I in 44/45. Primers 5′-TAC CAA AGG AAA TGC ATC ATT G-3′ and 5′-AAT TGG TTC CGG AGT CGC-3′ were used to characterize the insertion of pp65 in Del3. PCR products were analyzed by 100 V electrophoresis on a 1% agarose gel stained with ethidium bromide and imaged using Syngene PXi6 imager with GeneSys (v1.5.4.0) software. Gels from each experiment were processed in parallel. Uncropped and unprocessed scans of the gels have been supplied as Source Data files.

### Western Blot analysis

To characterize the CMV antigens expression of the sMVA viral stocks, CEF cells were seeded in 6-well plates, grown to ~80–90% confluency, and infected at 1 MOI with the corresponding sMVA viral stock. At 16–24 h post infection, cells were harvested and proteins were reduced and denatured in Laemmli buffer supplemented with 5% 2-Mercaptoetanol and boiled at 90 °C for 10 min. Proteins were then resolved on a 4–20% Mini-PROTEAN TGX gradient gel (BioRad) and transferred onto PVDF membrane. IE1-4nt was probed using an anti-IE1 mouse mAb (p63-27)^48^ at a dilution of 1:10. IE2-4nt and IE2-4nt-M361I were probed using an anti-IE2 mouse mAb (2.9.5)^49^ at a dilution of 1:100. Pp65 was probed using an anti-pp65 mouse mAb (28-103)^50^ at a dilution of 1:10. Vaccinia virus BR5 was probed using an anti-BR5 rat mAb (19C2)^51^ at a dilution of 1:20. Anti-mouse IgG,A,M pAb conjugated with horseradish peroxidase (A0412) was used as a secondary Ab at a dilution of 1:20000 for anti-IE1, -IE2 and -pp65 primary Abs. Anti-rat IgG pAb conjugated with horseradish peroxidase (A5795) was used as a secondary Ab at a dilution of 1:3000 for anti-BR5 primary Ab. Protein bands were visualized with SuperSignal Chemiluminescent Substrate (Thermo Fisher Scientific). Blots from each experiment were processed in parallel. Uncropped and unprocessed scans of the blots have been supplied as Source Data files.

### Animal models

The Institutional Animal Care and Use Committee (IACUC) of the Beckman Research Institute of City of Hope approved protocol 98004 assigned for this study. All study procedures were carried out in strict accordance with the recommendations in the Guide for the Care and Use of Laboratory Animals and the Public Health Service Policy on the Humane Care and Use of Laboratory Animals. Mice were kept on a 12-h light/12-h dark cycle, at 22–24 °C and 30–70% humidity, with ad libitum access to food and water. HLA-A*0201 H-2Dbβ2m double knockout (HLA-A2) transgenic mice on a C57BL/6 background^40^ were purchased from Charles River Laboratories and bred at the City of Hope Animal Research Center. Sixteen HLA-A2 mice between eight- and twelve-week-old were distributed in three groups: T10-F10 (*n* = 6), Triplex (*n* = 4) and s52 (*n* = 6). HLA-B*0702 H-2KbDb double knockout (HLA-B7) transgenic mice on a C57BL/6 background^41^ were obtained from F. Lemonnier (Institut Pasteur, France) and bred at the City of Hope Animal Research Center. Twelve seven-week-old HLA-B7 mice were distributed in three groups: T10-F10 (*n* = 4), Triplex (*n* = 4) and s52 (*n* = 4). HLA-A2 and B7 mice were immunized twice in 4 weeks interval by intraperitoneal route with 1 × 10^7^ PFU of T10-F10, Triplex or s52. Anesthesia was induced by placing the mice for 2–3 minutes in a chamber with 3–4% volume per volume (v/v) isoflurane. The experimental endpoint was 1 week post booster immunization when splenocytes for cellular immune analysis were collected and isolated by standard procedure after animals were humanely euthanized using carbon dioxide (CO2). Using a non-pre-charged chamber, CO2 was dispensed from a commercial cylinder with fixed pressure regulator. CO2 flow was maintained for over 60 seconds following respiratory arrest.

### ELISpot

T cell detection by IFNγ ELISpot assay was performed according to the manufacturer’s instructions (3321–2 A, Mabtech). Briefly, ELISpot PVDF plates (MSIPS4W10, Millipore) were pre-activated with 35% ethanol, coated with IFNγ-coating antibody and incubated overnight at 4–8 °C. Splenocytes (2 × 10^5^ if peptide-stimulated, 5 × 10^4^ if PMA/Ionomycin-stimulated) were added to duplicate wells and incubated overnight with 2 μg/mL peptides. Stimuli used were IE1, IE2 and pp65 peptide libraries as well as HLA-A2-restricted pp65- and IE1-specific immunodominant epitope peptides pp65 495–503 (NLVPMVATV) and IE1 316–324 (VLEETSVML) and HLA-B7-restricted pp65-specific immunodominant epitope peptide pp65 265-275 (RPHERNGFTVL). After 16–24 h, cells were removed from the wells, and IFNγ-detection antibody followed by streptavidin-ALP were added. Spots were developed using BCIP/NBT-plus (3650-10, Mabtech) and analyzed using CTL Analyzer immunoSpot plate reader. DMSO was used as negative control for splenocyte stimulation, and all values shown in Fig. 6 were DMSO-normalized.

### Sequencing

To characterize the integrity of the IE2-4nt gene sequence within the reconstituted sMVA vectors, CEF cells were seeded in 6-well plates, grown to ~80–90% confluency, and infected at 1 MOI with the corresponding sMVA viral stock. At 16–24 h post infection, cells were harvested, and DNA was extracted using the Quick-DNA Miniprep Plus Kit (ZYMO RESEARCH) according to the manufacturer’s instructions. The antigen sequence was PCR-amplified with Phusion polymerase (Thermo Fisher Scientific) using the same primers used for the characterization of the 44/45 insertion site (5′-TCC ATT GTA GAT TGT TGA CCG-3′ and 5′-ATA CAT ACC ATC GAC ATC CAT TAG C-3′), leading to amplification of the antigen gene sequence and flanking regions of the insertion site. After a 100 V electrophoresis on a 1% agarose gel stained with ethidium bromide, the NucleoSpin Gel and PCR clean-up kit (Macherey-Nagel) was used to purify the amplified PCR products. Eton Biosciences performed the Sanger sequencing of the purified DNA with the sequencing primers: 5′-TCC ATT GTA GAT TGT TGA CCG-3′, 5′-ATA CAT ACC ATC GAC ATC CAT TAG C-3′, 5′-ACT TCT TCA CCC TGT TCT TCC TC-3′, 5′-GTA AGA AAC CGC GCA AGA CC-3′, 5′-TCG CAA GAA GAA GAG CAA ACG-3’ and 5′-ATG CTT GTA ACG AAG GCG TC-3′.

### Statistics

Statistical analysis was performed using GraphPad Prism (v8.3.0). Two-way ANOVA with Tukey’s multiple comparison test was used for statistical evaluation after logarithmic transformation to calculate the significance between groups on the ELISpot results. Each mouse corresponded to one sample. Age and gender were not contemplated for the analysis.

### Reporting summary

Further information on research design is available in the Nature Research Reporting Summary linked to this article.

### Supplementary information

Data Set 1

Data Set 2

Data Set 3

Data Set 4

Data Set 5

Data Set 6

Reporting Summary

## Data Availability

The datasets generated during and/or analyzed during the current study are available from the corresponding author upon reasonable request.
